# CT Perfusion to Guide Placement of Invasive Cerebral Perfusion Monitor in Subarachnoid Hemorrhage Induced Vasospasm

**DOI:** 10.1155/2018/5162629

**Published:** 2018-04-04

**Authors:** Hosam Al-Jehani, Judith Marcoux, Kawthar Hadhiah, Faisal Alabbas, Mark Angle, Jeanne Teitelbaum

**Affiliations:** ^1^Department of Neurology and Neurosurgery, Montreal Neurological Institute and Hospital, McGill University, Montreal, QC, Canada; ^2^Department of Neurosurgery and Critical Care Medicine, King Fahad Hospital of the University, Imam Abdulrahman bin Faisal University, Al-Khobar, Saudi Arabia

## Abstract

**Background:**

Vasospasm is a challenging component of the subarachnoid hemorrhage “syndrome” that is unpredictable and very difficult to monitor using noninvasive or invasive monitoring technologies in neurocritical units.

**Methods:**

We describe the novel use of computerized tomography perfusion (CTP) imaging to choose proper targets for invasive cerebral blood flow monitors.

**Results:**

A total of 3 patients are included in this report. CTP parameters were used to generate points of interest to target using invasive cerebral monitoring of the cerebral blood flow and initiate vasodilator therapy and subsequently guide its weaning.

**Conclusions:**

CTP can be useful in localizing a specific anatomical target for invasive monitoring in subarachnoid hemorrhage patients suffering from vasospasm.

## 1. Introduction

Vasospasm is a devastating event, complicating as high as 50% of SAH with increased morbidity and mortality [1]. Most imaging modalities (such as cerebral angiogram, CT-angiogram, or transcranial Doppler) do not give continuous monitoring and therefore are typically used only if there is clinical suspicion of vasospasm. Several methods have been in use to detect vasospasm early on, prior to its clinical manifestation, including continuous transcranial Doppler (TCD) and electroencephalography (EEG) [2, 3]. More recently, invasive monitoring devices have been implemented, again in the effort to detect vasospasm early, including brain oxygen monitors and cerebral perfusion monitors [4, 5].

The major drawback of these devices is the small sampling volume, which precludes robust assessment of early changes potentially leading to vasospasm. Choosing a target has been done through standardized landmarks and insertion angles of a monitoring "bundle" in either frontal lobe depending on the seen or sometimes perceived burden of pathology. Another localizing strategy is to place the invasive cerebral monitor in the vicinity of the lesion seen on imaging in cases of focal injury with recent evidence of improvement in outcome [6]. This strategy proves less reliable and potentially futile in a dynamic disease such as SAH and vasospasm with an essentially normal brain parenchyma on CT scans until the development of vasospasm and progression to hypodensities, at which point the futility of treatment is a major concern.

Recent refinement in computed tomography perfusion (CTP) imaging led to more utilization of this technology in vasospasm [7]. Several parameters have been debated as to their ability and reliability of vasospasm detection. Of those, time-to-peak (TTP) has gained popularity as a surrogate for assessment of cerebral autoregulation, the impairment of which is thought to be conducive to vasospasm [8].

We describe a series of cases in which we used CT perfusion parameters, especially the time-to-peak, to guide localization of the Bowman Perfusion Monitor (BPM) insertion in patients suspected to have vasospasm.

## 2. Case Summaries

### 2.1. Case  1

**Figure 1:**
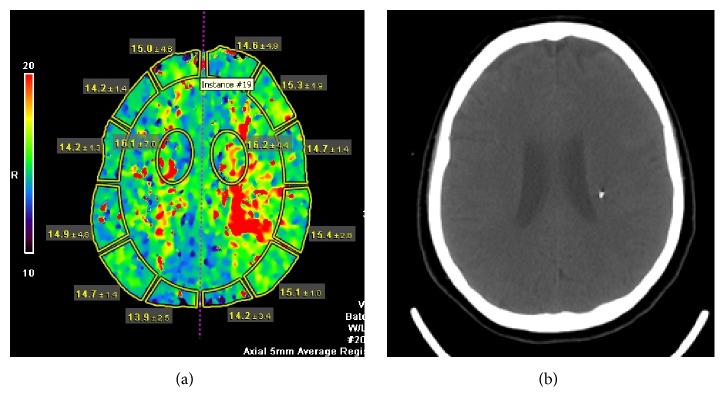
(a) prolongation of the TTP signal in the left central Rolandic white matter. (b) A plain CT scan showing the tip of the BPM in the area of the prolongation of the TTP.

A 34-year-old woman presented with abrupt onset headache and lethargy attributable to an acute SAH secondary to a ruptured P-1 aneurysm. She received standard therapy for SAH patients and had successful coiling of the aneurysm. On postbleeding day 4, while hemodynamically stable, she developed a new drift in the right upper extremity that progressed to grade 3/5 motor weakness with facial weakness within 30 minutes. A repeat plain CT showed no hydrocephalus. The CT perfusion showed no derangement of the cerebral blood volume (CBV) or the cerebral blood flow (CBF) with a slightly prolonged mean transit time (MTT) denoting intact brain tissue. The time-to-peak (TTP), on the other hand, was significantly prolonged in the left central Rolandic subcortical region, consistent with the clinical finding (Figure 1). A Bowman Perfusion Monitor (BPM, Hemedex, MA, USA) was inserted at the bedside, using free-hand technique aiming at the point of interest generated by the CT perfusion TTP scan. The cerebral blood flow (CBF) measured at this time was 14 cc/100 g/min, denoting severe hypoperfusion (normal level is above 25 cc/100 g/min). The patient was started on the MNH-milrinone protocol for the treatment of vasospasm [9]. Within 1 hour, the patient did not improve, necessitating endovascular vasodilator therapy, during which she received 2 mcg of milrinone over 3 minutes with immediate vasodilation of the vasospastic segment. The CBF reading improved to the 50's with the maintenance of milrinone. On 2 occasions, weaning the milrinone infusion was associated with an asymptomatic but significant drop of the measured CBF to low 20's, which delayed the weaning for 24–48 hours each time, likely related to recoil of angioplastied vessel mitigated clinically by the milrinone effect. The total monitoring period was 9 days, after which milrinone was stopped and CBF measurement ranged from 40 to 50 and the patient did not have any residual motor deficits for an additional 48 hours.

### 2.2. Case  2

A 46-year-old male suffered a grade 4 SAH, with clinical evidence of a rebleed prior to arrival, associated with a left frontal hematoma. Angiography showed a small blister aneurysm in the ophthalmic segment of the left internal carotid artery for which the patient was submitted to a craniotomy and clipping of the aneurysm. CTP was done postoperatively showing no asymmetry in the perfusion of the 2 cerebral hemispheres. On postadmission day 8, the patient developed new right-sided weakness; the noncontrast CT scan excluded the presence of hypodensities other than the perihematoma edema around the left frontal bleed. CT angiogram showed evidence of vasospasm in the left A1 and anterior-communicating artery with no evidence of vasospasm in the left MCA. The CTP maps showed no evidence of infarction as there was no CBF/CBV other than the artifact of the hematoma. Although subtle, there was a prolongation of the TTP map in the left central white matter, which corresponded to the patient's deficit. That point was chosen to be the target for the cerebral perfusion monitor, which was inserted using a free-hand technique. After insertion, CT scan confirmed the proper targeting of the point of interest generated by the CTP-TTP map. The initial CBF was 16 and it improved to high 30's after the milrinone protocol was instituted. The patient was weaned in due course and the follow-up CT scan demonstrated no hypodensities other than that related to the left frontal hematoma.

### 2.3. Case  3

A 67-year-old female suffered a grade 4 SAH from a ruptured PCOM aneurysm, which was uneventfully coiled. She had a complicated course in the NICU with a new onset atrial fibrillation and labile hypertension. She deteriorated after admission day 4 in the form of reduced level of response, but no lateralizing deficit was detected. A plain CT scan showed evidence of a new hypodensity in the right frontal region. A CTP confirmed that this hypodensity corresponded to an area of cerebral infarction by CBV/CBF maps. Interestingly, the central white matter in the right hemisphere showed prolonged TTP. We chose that as the target for the cerebral perfusion monitor. The initial CBF was 22, which improved marginally to 26 after a milrinone protocol was instituted. The trend in the first 12 hours of monitoring was that of a stable CBF reading but the brain temperature was noted to be increasing gradually from 36.6 to 38.1 without systemic fever detected by the nursing team and a stable CBF recording. This was followed after few hours by a drop of the CBF to 15 coinciding with significant worsening of the patient's level of consciousness. Another bolus of milrinone and an increased dose failed to improve the CBF, which continued to drop to as low as 6, being in the zone of frank cerebral ischemia. A new CT scan showed the development of several hypodensities bilaterally, including the area that was chosen for monitoring. Further increases of milrinone were not tolerated due to cardiac decompensation, and further escalation of therapy was deferred.

## 3. Discussion

Invasive cerebral monitoring of cerebral homeostatic functions is gaining popularity in the field of neurocritical care. It can provide anticipatory data in the evolution of cerebral insults prior to clinical deterioration, permitting, at least in theory, a better cerebral protection from secondary injury and improvement in outcome. One of the major concerns with these modalities of monitoring is the small sampling size within the injured or diseased brain. This and the usual placement in the frontal lobe to be as close to the known land marks of ICP monitoring insertion as possible result in a forced sampling strategy. This could be useful in a brain insult, where there is a focal lesion in or near the frontal area. On the contrary, in a dynamic or unpredictable disease such as vasospasm, choosing predetermined target might lead to missed monitoring opportunity because of lack of useful information as the deterioration might be occurring in another lobe or the other hemisphere.

Our cases illustrate the useful utilization of noninvasive CTP imaging data to generate points of interest for invasive monitoring. This combination overcomes the random field invasive monitoring, which has a great potential to reduce the accuracy of the monitoring process or missampling the “real” target area. In all 3 patients, none of the probes inserted were inserted in the frontal region, which would have been the usual target for the insertion of the monitoring bundle. In one of the patients (Case  3), if the probe had been inserted in the frontal lobe, it would have landed in an infarcted area, potentially skewing the management escalation of the patient. CTP was instrumental in detecting areas of potential derangement of cerebral autoregulation and vasospasm, which are out of the usual frontal zone of insertion of the monitoring bundle. This will allow for the ideal application of this technology in the setting of SAH, as it could detect event leading up to the clinical vasospasm, such as perturbed autoregulation, at a much earlier time frame [10]. With refinement of the technology and better understanding of its output, its advantage and clinical utility will increase. For example, the proper targeting allowed more reliable information that incurred an escalation, maintenance, and withdrawal of care in our patients. Because the potential targets would be variable based on CTP maps, free-hand insertion of invasive monitoring poses an accuracy concern. This issue can be improved and potentially resolved by the use of Neuronavigation systems abbreviated to suit the needs of “points-care” setup as in this case, the ICU.

In conclusion, utilization of data generated by noninvasive radiological imaging such as CTP would potentially increase our accuracy of targeting points of interest in monitoring for vasospasm in subarachnoid hemorrhage patients and guide their therapy.
